# GRANDMOTHER CAREGIVERS’ RESILIENCE, MINDFULNESS, AND RESOURCEFULNESS AND MCCUBBIN’S MODEL OF FAMILY STRESS

**DOI:** 10.1093/geroni/igad104.0404

**Published:** 2023-12-21

**Authors:** Carol Musil, Alexandra Jeanblanc, Jaclene Zauszniewski, Christopher Burant

**Affiliations:** Case Western Reserve University, Cleveland, Ohio, United States; Case Western Reserve University, Cleveland, Ohio, United States; Case Western Reserve University, Cleveland, Ohio, United States; Case Western Reserve University, Cleveland, Ohio, United States

## Abstract

Grandmothers raising grandchildren face numerous challenges that are reflected in their levels of depressive symptoms and perceived family functioning. Using McCubbin’s Resiliency Model of Family Stress, Adjustment and Adaptation, we examine how demographics, family demands (intra-family strains), mediators of problem-solving/coping (resilience, mindfulness and resourcefulness), resources (support) and situational appraisals (stress and reward) affect a) depressive symptoms and b) family functioning. Our participants were a nationwide sample of 300 American grandmothers raising grandchildren who participated in an online research study. Our regression model for general family functioning had an adjusted R2 of .44. Better self-appraisal of family functioning was significantly associated with younger participant age, identifying as white, higher levels of resourcefulness, fewer reported intra-family strains, more subjective social support and instrumental support. Situational appraisals, the problem solving/coping factors of resilience and mindfulness, employment status, and marital status did not significantly predict family functioning. Depressive symptoms, as measured by the CES-D scale, had an adjusted R2 of .46. Participants who were married, had fewer intra-family strains, higher levels of mindfulness and resilience, higher subjective support, and who appraised greater reward from caregiving to grandchildren had lower levels of depressive symptoms. There was no effect from caregiving stress, resourcefulness, employment status, or race. These analyses show the continued utility of McCubbin’s model, despite different patterns of predictors of family functioning and depressive symptoms. This further develops our prior work showing that resilience, mindfulness, and resourcefulness are correlated but independent theoretical constructs as they interact very differently with our two outcomes.

